# Characteristics and Outcomes of Patients Receiving a Second Rescue Valve During Transcatheter Aortic Valve Implantation

**DOI:** 10.1016/j.shj.2023.100231

**Published:** 2023-11-14

**Authors:** Henrik Bjursten, Sasha Koul, Pétur Pétursson, Jacob Odenstedt, Henrik Hagström, Jenny Backes, Niels Erik Nielsen, Andreas Rück, Jan Johansson, Stefan James, Magnus Settergren, Matthias Götberg, Troels Yndigen

**Affiliations:** a Department of Cardiothoracic Surgery, Lund University, Skåne University Hospital, Lund, Sweden; b Department of Cardiology, Clinical Sciences Lund, Lund University, Skåne University Hospital, Lund, Sweden; c Department of Cardiology, Sahlgrenska University Hospital, Gothenburg, Sweden; d Department of Public Health and Clinical Medicine, Heart Centre, Umeå University, Umeå University Hospital, Umeå, Sweden; e Department of Cardiothoracic and Vascular Surgery, Orebro University Hospital, Örebro, Sweden; f Department of Cardiology, Heart Centre, University Hospital, Linköping, Sweden; g Department of Cardiology, Karolinska University Hospital and Karolinska Institute, Stockholm, Sweden; h Department of Cardiology, Blekinge Hospital, Karlskrona, Sweden; i Department of Medical Sciences and Uppsala Clinical Research Center, Uppsala University, Uppsala, Sweden

**Keywords:** Complication, Outcome, Survival, TAVI

## Abstract

**Background:**

Transcatheter aortic valve implantation (TAVI) has become a safe procedure. However, complications occur, including uncommon complications such as valve malposition, which requires the implantation of an additional rescue valve (rescue-AV). The aim was to study the occurrence and outcomes of rescue-AV in a nationwide registry.

**Methods:**

The Swedish national TAVI registry was used as the primary data source, where all 6706 TAVI procedures from 2016 to 2021 were retrieved. Nontransfemoral access and planned valve-in-valve were excluded. In total, 79 patients were identified as having had a rescue-AV, and additional detailed data were collected for these patients. This dataset was analyzed for any characteristics that could predispose patients to a rescue-AV. The outcome of patients receiving rescue-AV also was studied.

**Results:**

Of the 5948 patients in the study, 1.3% had a rescue-AV. There were few differences between patients receiving 1 valve and rescue-AV patients. For patients receiving a rescue-AV, the 30-day mortality was 15.2% compared to 1.6% in the control group. A poor outcome after rescue-AV was often associated with a second complication; for example, stroke, need for emergency surgery, or heart failure. Among the patients with rescue-AV who survived at least 30 days, landmark analyses showed similar survival rates compared to the control group.

**Conclusions:**

Among TAVI patients in a nationwide register, rescue-AV occurred in 1.3% of patients. The 30-day mortality in patients receiving rescue-AV was high, but long-term outcome among 30-day survivors was similar to the control group.

## Graphical abstract

**Figure:**
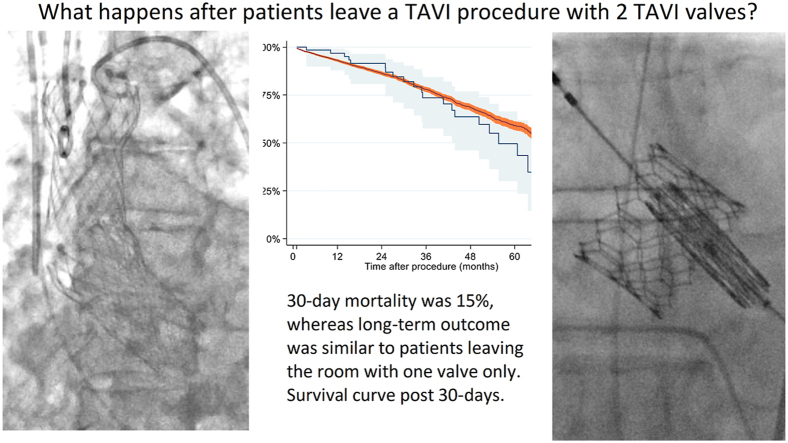

## Introduction

Transcatheter aortic valve implantation (TAVI) has revolutionized the care of patients with aortic stenosis, and indications have expanded to patients who have traditionally been treated with surgical aortic valve replacement (SAVR).^1^, ^2^, ^3^ Recent studies have shown similar or improved short-term and medium-term outcomes for TAVI compared with SAVR.^2^^,^^3^

Still, TAVI is associated with uncommon complications not seen in surgery, such as femoral complications, coronary artery occlusion, and mechanical complications caused by the delivery system.^4^ In addition, the TAVI valve may not be delivered in the exact intended position or may even end up in an ectopic position after valve embolization.^5^^,^^6^

A recent study described the occurrence of TAVI valve embolization in a large cohort and showed a reasonable short-term outcome.^6^ These findings were confirmed in a subsequent smaller study.^5^ A less severe complication is valve malpositioning where the valve is still anchored in the aortic valve but positioned too high or too low. This condition may require a second valve to secure the first or to reduce paravalvular leakage.^5^^,^^7^ All these studies have a mix of first-generation and second-generation valves and report up to 1-year follow-up with an acceptable outcome.

Therefore, the present study aimed to investigate cases where the patient left the procedure with more than 1 valve, either due to valve embolization or malpositioning, in a cohort with a vast majority of second-generation valves. The study is a nationwide cohort study comprising all TAVIs performed in Sweden during a 6-year period. The main objective was to compare the characteristics and long-term outcomes of patients needing a rescue additional valve (rescue-AV) to a control group of TAVI patients during the same time period.

## Methods

### Study Design

**Figure 1:**
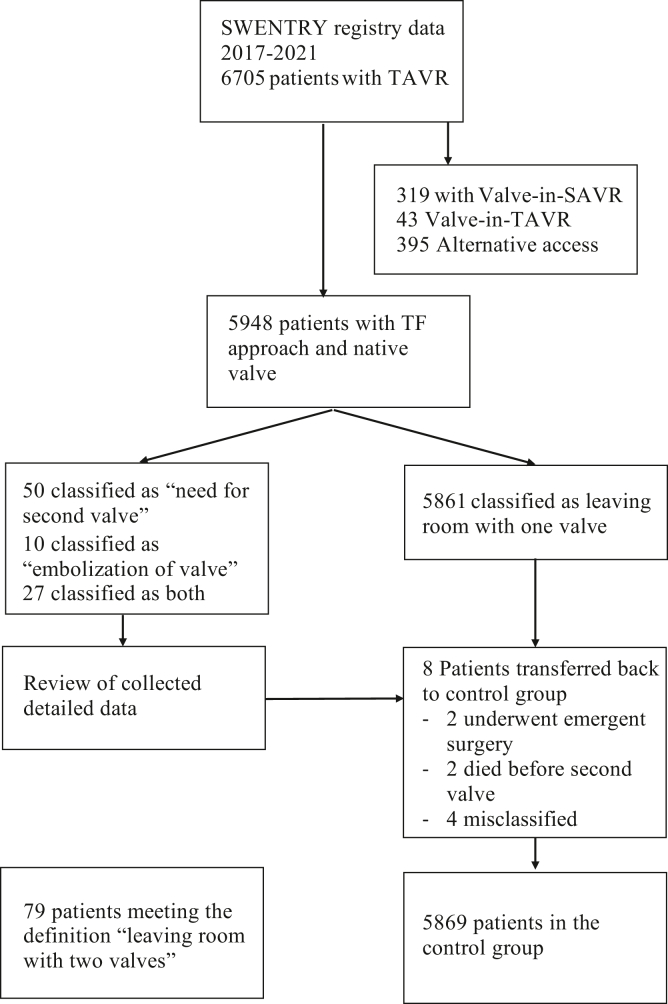
**Flow chart of study design. Flow chart depicting study patient selection from SWENTRY registry**. Abbreviation: SAVR, surgical aortic valve replacement; SWENTRY, SWEdish traNscatheter cardiac intervention regisTRY; TAVR, transcatheter aortic valve replacement; TF, transfemoral.

This is a retrospective nationwide follow-up study of all patients who underwent TAVI in Sweden from January 2016 to December 2021. The initial data source was the National TAVI registry SWENTRY (SWEdish traNscatheter cardiac intervention regisTRY), which is part of the SWEDEHEART registry (Swedish Web-system for Enhancement and Development of Evidence-based Care in Heart Disease Evaluated According to Recommended Therapies). It contains information on all TAVI procedures performed in the country and has a high degree of accuracy and coverage.^8^, ^9^, ^10^ The registries are coupled to the Swedish Population Registry with a 100% catchment of mortality. During the study period, a total of 6706 patients received TAVI. The rescue-AV study group was defined as patients “leaving the procedure with more than one valve”. From this study group, 1 patient was excluded due to missing data, 362 patients were excluded because of planned Valve-in-Valve, and 395 patients were excluded due to nontransfemoral access (Figure 1). From this dataset, we selected patients who were marked as “Need for second valve” (50 patients), “valve embolization" (10 patients), or both (27 patients). We then contacted all 8 centers performing TAVI in Sweden and requested a review of these 87 patients and detailed information through a structured questionnaire. Eight of these 87 patients were then excluded from the rescue-AV study group (4 patients had the first valve retrieved before implantation of the second valve, 2 patients with embolized valve suffered instant death before receiving a second valve, and 2 patients with an embolized valve went to surgery); these patients were included in the control group instead. The control group consisted of all patients who underwent TAVI with only 1 valve via transfemoral access during the same period. The study was approved by the National Ethical Review Board in Sweden (registration numbers 2017/995 and EPN 2019/0584). The study is reported according to the STROBE guidelines.^11^

### Statistical Analysis

Categorical data are presented as numbers (percent of the population), and continuous variables are presented as mean ± standard deviation. For comparisons between groups, the Chi-square test was used for categorical data and Student’s t-test for continuous data. Logistic regression was used to identify risk factors associated with rescue-AV. Propensity score matching was employed using the nearest neighbor method with a 1:2 matching on the following variables: age, gender, serum creatinine, hypertension, diabetes, peripheral vascular disease, NYHA class, aortic valve area (AVA), bicuspid valve (Supplemental Material). Kaplan–Meier curves were used to show survival over time, and survival between groups was tested using a Log-rank test. A separate analysis of survival past 30 days also was performed (Landmark analysis). Statistical testing was performed with Stata ver. 17 (StataCorp, College Station, TX, USA)

## Results

Seventy-nine (1.3%) of 5948 patients had a rescue-AV according to the definition of this study (Figure 1). Demographics were similar between the study group (rescue-AV) and the control group. However, there was a significant difference in aortic valve area, where the control group had an aortic valve area (AVA) of 0.70 ± 0.19 cm^2^ and the rescue-AV group had an AVA of 0.63 ± 0.19 cm^2^ (*p* = 0.003, Table 1) and almost twice as many were labeled as urgent procedures (20.3 vs. 11.3%, *p* = 0.013, Table 1). Only a small fraction in both groups had a first-generation valve (0.5%). During the procedure, the rescue-AV group had almost twice the fluoroscopy time (1975 ± 1034 ​seconds vs. 1024 ± 617 ​seconds, *p* < 0.001). They also required almost double the amount of contrast (111.6 ± 62.2 ml vs. 60.7 ± 41.8 ml, *p* < 0.001, Table 2). In the rescue-AV group, 72% (57 of 79) had a self-expandable valve, whereas in the control group, 54% (3164 of 5869) had self-expandable valve (*p* < 0.001, Table 2).

**Table 1:** Demographics

Demographics	All (n = 5948)	Control (n = 5869)	Rescue-AV (n = 79)	OR (95% CI)
Age (years)	81.1 ± 6.8	81.1 ± 6.7	81.5 ± 7.5	1.00 (0.98-1.04)
Female gender	2738 (46.0%)	2702 (46.0%)	36 (45.6%)	0.98 (0.61-1.57)
Height (cm)	168.7 ± 9.5	168.8 ± 9.5	167.7 ± 9.1	0.99 (0.97-1.01)
Weight (kg)	76.6 ± 16.3	76.6 ± 16.2	75.1 ± 20.6	0.99 (0.98-1.00)
Body mass index	26.8 ± 5.0	26.8 ± 5.0	26.4 ± 5.7	0.98 (0.94-1.03)
Serum creatinine (μmol/L)	100.6 ± 59.3	100.7 ± 59.6	96.1 ± 32.5	1.00 (0.99-1.00)
Hypertension	4591 (77.2%)	4526 (77.1%)	66 (82.3%)	1.38 (0.76-2.67)
Diabetes	1531 (25.7%)	1512 (25.8%)	19 (24.1%)	0.91 (0.51-1.55)
Previous cardiac surgery	693 (11.7%)	682 (11.6%)	11 (13.9%)	1.23 (0.58-2.35)
Recent myocardial infarction	217 (3.6%)	214 (3.6%)	3 (3.8%)	1.04 (0.21-3.20)
Previous PCI	1587 (26.7%)	1572 (26.8%)	15 (19.0%)	0.64 (0.33-1-14)
COPD	948 (15.9%)	935 (15.9%)	13 (16.5%)	1.03 (0.52-1.91)
Previous stroke	663 (11.1%)	649 (11.1%)	14 (17.7%)	1.73 (0.89-3.14)
Critical preoperative state	129 (2.2%)	126 (2.1%)	3 (3.8%)	1.79 (0.36-5.58)
Peripheral vascular disease	736 (12.4%)	724 (12.3%)	12 (15.2%)	1.27 (0.62-2.39)
Reduced mobility	745 (12.5%)	738 (12.6%)	7 (8.9%)	0.67 (0.26-1.47)
Atrial fibrillation	2180 (36.7%)	2155 (36.7%)	25 (31.6%)	0.80 (0.47-1.30)
Steroid treatment	417 (7.0%)	412 (7.0%)	5 (6.3%)	0.89 (0.28-2.19)
History of malignancy	606 (10.2%)	594 (10.1%)	12 (15.2%)	1.59 (0.78-2.99)
Pacemaker bearer	609 (10.2%)	599 (10.2%)	10 (12.7%)	1.27 (0.58-2.50)
Urgency				
Elective procedure	5224 (87.8%)	5162 (88.0%)	63 (78.5%)	Ref
Urgent procedure	679 (11.4%)	663 (11.3%)	16 (20.3%)	2.01 (1.15-3.50)
Acute procedure	1173 (19.7%)	1167 (19.9%)	6 (7.6%)	1.90 (0.26-13.9)
NYHA class				
NYHA I	159 (2.6%)	157 (2.7%)	2 (2.5%)	0.82 (0.20-3.42)
NYHA II	1173 (19.7%)	1167 (19.8%)	6 (7.6%)	0.33 (0.14-0.78)
NYHA III	4029 (67,7%)	3968 (67.6%)	61 (77.2%)	Ref
NYHA IV	581 (9.8%)	571 (9.7%)	10 (12.6%)	1.14 (0.582.23)
LVEF				
LVEF normal	3899 (65.6%)	3845 (65.5%)	55 (68.4%)	Ref
LVEF slightly depressed	799 (13.4%)	794 (13.5%)	5 (6.3%)	0.45 (0.18-1.12)
LVEF moderately depressed	801 (13.5%)	789 (13.4%)	12 (15.2%)	1.08 (0.58-2.03)
LVEF severely depressed	429 (7.2%)	421 (7.2%)	8 (10.1%)	1.35 (0.64-2.86)
Bicuspid Valve	453 (7.7%)	444 (7.6%)	9 (12.1%)	1.57 (0.68-3.18)
Mean annular diameter	24.6 ± 2.3	24.6 ± 2.3	24.6 ± 2.5	1.01 (0.91-1.11)
Mean aortic gradient (mmHg)	47.4 ± 14.1	47.4 ± 14.1	47.4 ± 14.6	1.00 (0.98-1.01)
AVA (cm^2^)	0.71 ± 0.17	0.71 ± 0.16	0.64 ± 0.15	0.09 (0.02-0.39)

Comparison between control group (patients with 1 TAVI) and rescue-AV group (patients receiving more than 1 valve due to malpositioning or embolization).

Abbreviations: AVA, aortic valve area; CI, confidence interval; COPD, chronic obstructive pulmonary disease; LVEF, left ventricular ejection fraction; NYHA, New York Heart Association; OR, odds ratio.

**Table 2:** Valve distribution

Valve Model	All (5948)	Control (5869)	Rescue-AV (79)
Abbott Navitor	38	38	
Abbott Portico	218	205	13
Boston Acurate neo	997	987	10
Boston Acurate neo2	440	437	3
Boston Acurate TA	1	1	
Boston Lotus	77	75	2
Boston Lotus Edge	45	45	
Edwards Sapien 3	1600	1587	13
Edwards Sapien 3 Ultra	990	983	7
Edwards Sapien and Sapien XT	4	4	
Medtronic CoreValve	23	18	5
Medtronic Evolut PRO	349	342	7
Medtronic Evolut PRO Plus	1	1	
Medtronic Evolut R	1154	1135	19
Meril MyVal	11	11	
SEV	3232	3175	57
BEV	2716	2694	22

Valve distribution for the entire group, control group and rescue-AV (patients receiving more than 1 valve due to malpositioning or embolization).

Boston Lotus is included in the BEV group. Distribution of SEV between groups yields a *p*-value *p* < 0.001.

Abbreviations: BEV, balloon expanding valve; SEV, self-expanding valve.

At 30-day follow-up, the rescue-AV group had a higher mortality (15.2 vs. 1.6%, *p* < 0.001), a larger proportion of stroke (15.2 vs. 1.5%, *p* < 0.001) and minor stroke (1.3 vs. 0.1%, *p* = 0.01), more patients in need of circulatory support (2.5 vs. 0.1%, *p* < 0.001), more unplanned general anesthesia (3.8 vs. 0.9%, *p* = 0.01), a larger number of myocardial infarctions (1.3 vs. 0.2%, *p* = 0.02), more postprocedural infections (6.3 vs. 2.2%, *p* = 0.02), and more new renal replacement therapy (1.3 vs. 0.1%, *p* < 0.001). For the 70 patients in the rescue-AV group who had an echocardiographic assessment, there was a higher frequency of moderate paravalvular regurgitation (7.5 vs. 2.8%, *p* = 0.01) as well as severe paravalvular regurgitation (1.3 vs. 0.1%, *p* = 0.001). There were no differences in postoperative peak gradient or creatinine at discharge (Table 3).

**Table 3:** Outcome data

Intraprocedural data	Entire cohort			Propensity matched cohort		
	Control (n = 5869)	Rescue-AV (n = 79)	*p* value	Control (n = 140)	Rescue-AV (n = 70)	*p* value
Contrast (ml)	60.7 ± 41.8	112.3 ± 62.3	<0.001	61.3 ± 49.3	113.3 ± 63.0	<0.001
Fluoroscopy time (min)	17.1 ± 10.3	33.1 ± 17.2	<0.001	18.5 ± 11.0	29.7 ± 17.1	<0.001
Predilatation	3477 (59.2%)	52 (64.6%)	0.3442	83 (59.3%)	43 (61.4%)	0.7650
Postdilatation	1414 (24.1%)	24 (30.4%)	0.1951	37 (26.4%)	18 (25.7%)	0.9116

Outcome 30 d	Control (n = 5869)	Rescue-AV (n = 79)	*p* value	Control (n = 140)	Rescue-AV (n = 70)	*p* value
Death within 30 d	95 (1.6%)	12 (15.2%)	<0.001	2 (1.4%)	12 (17.1%)	<0.001
Acute cardiac surgery	9 (0.2%)	0 (0.0%)	0.7276	0 (0.0%)	0 (0.0%)	1.0000
Circulatory support	5 (0.1%)	2 (2.5%)	<0.001	0 (0%)	2 (2.9%)	0.0447
Unplanned general anesthesia	52 (0.9%)	3 (3.8%)	0.0072	1 (0.7%)	3 (4.3%)	0.0742
Bleeding	180 (3.1%)	6 (8.5%)	0.0117	4 (2.9%)	6 (9.7%)	0.0419
Major stroke	79 (1.3%)	12 (15.2%)	<0.001	2 (1.4%)	11 (15.7%)	<0.001
Minor stroke	8 (0.1%)	1 (1.3%)	0.0103	1 (0.7%)	1 (1.4%)	0.6153
Myocardial infarction	9 (0.2%)	1 (1.3%)	0.0165	0 (0%)	1 (1.4%)	0.1563
Vascular complication	86 (1.5%)	3 (3.8%)	0.0900	1 (0.7%)	3 (4.3%)	0.0742
Infection needing treatment	129 (2.2%)	5 (6.3%)	0.0140	1 (0.7%)	5 (7.1%)	0.0084
New RRT	4 (0.1%)	1 (1.3%)	<0.001	0 (0%)	1 (1.4%)	0.1563
New permanent pacemaker	418 (7.1%)	7 (8.9%)	0.5512	9 (6.4%)	7 (10%)	0.1563
Creatinine at discharge	96.3 ± 63.3	89.0 ± 30.6	0.3365	84.7 ± 24.6	86.9 ± 29.3	0.5872
AI grade			<0.001			0.4601
0	3144 (53.6%)	35 (44.3%)	0.1010	67 (47.9%)	30 (42.9%)	
I	2452 (41.8%)	27 (34.2%)	0.1734	62 (44.3%)	25 (35.7%)	
II	166 (2.8%)	6 (7.6%)	0.0120	9 (6.4%)	5 (7.1%)	
III	5 (0.1%)	1 (1.3%)	0.0010	0 (0%)	0 (0%)	
Unknown	102 (1.7%)	10 (12.7%)	<0.001	2 (1.4%)	10 (14.3%)	
Peak gradient (mmHg)	18.6 ± 8.6	18.2 ± 7.4	0.7271	18.4 ± 7.8	16.6 ± 6.6	0.1899
Mean gradient (mmHg)	10.4 ± 5.2	9.8 ± 3.9	0.4210	9.8 ± 3.9	9.8 ± 4.0	0.9903

Comparison of outcome between control groups (patients with 1 TAVI) and rescue-AV group (patients receiving more than 1 valve due to malpositioning or embolization) for both entire cohort and propensity score matched cohort.

Abbreviations: AI, aortic insufficiency (no differentiation between central and paravalvular); RRT, renal replacement therapy.

**Figure 2:**
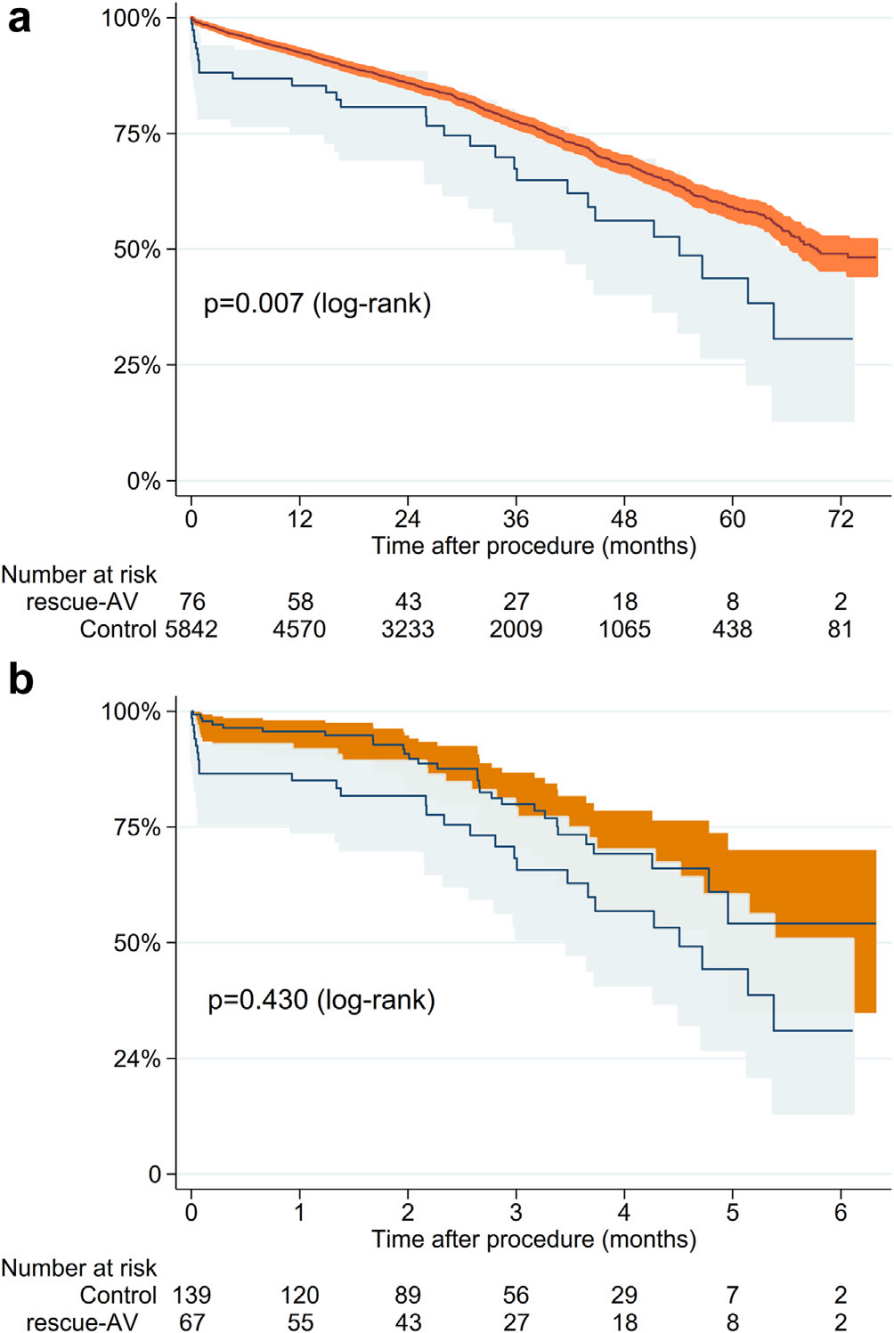
Overall survival for the groups. Kaplan–Meier curve depicting survival for the rescue-AV (patients receiving more than 1 valve due to malpositioning or embolization) for the entire cohort (a) and propensity score cohort (b). Log-rank test showed a significant difference with *p* = 0.007 (entire cohort) and *p* = 0.027 (propensity score cohort).

**Figure 3:**
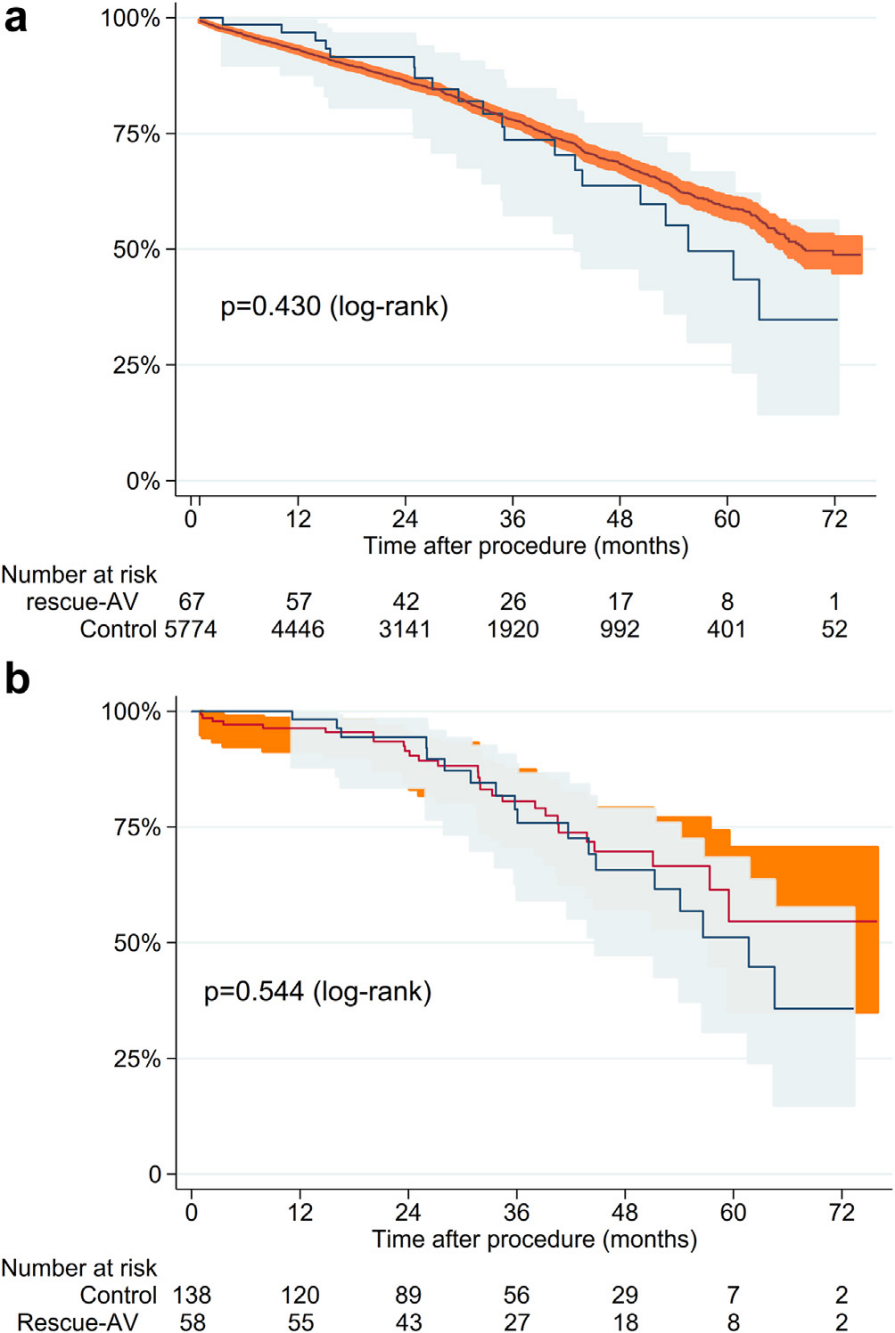
Landmark analysis after 30 days. Kaplan–Meier curve depicting survival for the rescue-AV group (patients receiving more than 1 valve due to malpositioning or embolization after 30 days) for entire cohort (a) and propensity score cohort (b). Log-rank test showed a nonsignificant difference with *p* = 0.430 (entire cohort) and *p* = 0.544 (propensity score cohort).

Patients were followed for survival with a mean of 880 days (range 124-2309 days). The high 30-day mortality in the rescue-AV group affected the long-term survival negatively compared to the control group (*p* = 0.007, Figure 2). However, when a landmark analysis was performed looking at survival past 30 days, no significant difference was observed between the 2 groups (*p* = 0.430, Figure 3).

A propensity scored cohort was created, that managed to match 70 rescue-AV with 140 patients who left the room with 1 valve. In this analysis, rescue-AV had a 17.1% 30-day mortality compared to 1.4% for the control group (*p* < 0.001, Table 3). The rescue-AV cohort had more stroke (15.7 vs. 1.4%, *p* < 0.001), needed more circulatory support (2.9 vs. 0%, *p* = 0.05) and had more infections (7.1 vs. 0.7%, *p* = 0.008). Kaplan–Meier survival curves showed high initial mortality for the rescue-AV group, but the 30-day landmark analysis showed a similar long-term survival compared to the control group (Figure 3) among those who survived to 30 days.

The retrospective data capture for rescue-AV patients revealed that 11% had a bicuspid valve, 97% were in a stable hemodynamic situation before the procedure, 47% had their first valve in the annulus, 36% had embolized to the aorta, and 17% had embolized to the ventricle (Table 4). The majority (95%) received the second valve during the same procedure. Only 10% needed circulatory support after the procedure (3% had extracorporeal membrane oxygenator (ECMO) and 7% had inotropic support). The stroke rate was high, with 9% having had a major stroke and 8% having had a minor stroke.

**Table 4:** Detailed outcome

Characteristic	All (n = 79)	Anchored in annulus (n = 50)	Embolized valve (n = 29)	Survived 30 d (n = 67)	Died within 30 d (n = 12)	*p* value 30-d mortality
Valve Morphology						
BAV Sievers 0	2 (3%)	1 (2%)	1 (3%)		2 (17%)	0.064∗
BAV Sievers 1	7 (9%)	6 (12%)	1 (3%)		1 (8%)	
Tricuspid	70 (89%)	43 (86%)	27 (93%)		9 (75%)	
Hemodynamic condition						
Stable	77 (97%)	48 (96%)	29 (100%)		11 (92%)	
Low BP (SBP<100)	1 (1%)	1 (2%)	0 (0%)		0 (0%)	
Vasopressor	1 (1%)	1 (2%)	0 (0%)		1 (8%)	
First Valve						
Accurate	12 (15%)	8 (16%)	4 (14%)		0 (0%)	0.320
Evolut	34 (43%)	23 (46%)	11 (38%)		5 (42%)	
Lotus	4 (5%)	4 (8%)	0 (0%)		0 (0%)	
Portico	13 (16%)	7 (14%)	6 (21%)		3 (25%)	
Sapien 3	16 (20%)	8 (16%)	8 (28%)		4 (33%)	
Embolization of the first valve						
No embolization	50 (63%)	50 (100%)	0 (0%)		7 (58%)	0.312†
To the aorta	25 (32%)	0 (0%)	25 (86%)		4 (33%)	
To the ventricle	4 (5%)	0 (0%)	4 (14%)		1 (8%)	
Second valve						
Evolut	36 (46%)	25 (50%)	11 (38%)		5 (45%)	
Lotus	2 (3%)	2 (4%)	0 (0%)		0 (0%)	
Portico	12 (15%)	7 (14%)	5 (17%)		3 (27%)	
Sapien 3	28 (35%)	16 (32%)	12 (41%)		3 (27%)	
Unknown	1 (1%)	0 (0%)	1 (3%)			
Placement of the first valve at the end of the procedure						
Anchored well in the annulus	37 (47%)	37 (74%)	0 (0%)		4 (33%)	0.629
Ascending aorta with some anchoring	13 (16%)	13 (26%)	0 (0%)		3 (25%)	
Ascending aorta with no anchoring	18 (23%)	0 (0%)	18 (62%)		4 (33%)	
Descending aorta	7 (9%)	0 (0%)	7 (24%)		1 (8%)	
Left ventricle	4 (5%)	0 (0%)	4 (14%)		0 (0%)	
Circulatory support after the procedure						
ECMO	2 (3%)	1 (2%)	1 (3%)		2 (17%)	
Inotropic support	6 (7%)	5 (10%)	1 (3%)		3 (25%)	
No	71 (90%)	44 (88%)	27 (93%)		7 (58%)	
Cardiac surgery						
No	76 (96%)	48 (96%)	28 (97%)		9 (75%)	
Emergent (same day)	2 (3%)	2 (4%)	0 (0%)		2 (17%)	
Nonmergent (within a month)	1 (1%)	0 (0%)	1 (3%)		1 (8%)	
Discharge data						
Stroke						
Major stroke	7 (9%)	1 (2%)	6 (21%)		5 (56%)	
Minor stroke	6 (8%)	1 (2%)	5 (17%)		0 (0%)	
No	61 (82%)	43 (96%)	18 (62%)		4 (44%)	
AI grade						
0	37 (51%)	21 (46%)	16 (59%)	33 (50%)		
I	29 (40%)	18 (39%)	11 (41%)	26 (39%)		
II	7 (10%)	7 (15%)	0 (0%)	7 (11%)		
III	0 (0%)			0 (0%)		
LVEF at discharge						
>50%	55 (70%)	32 (64%)	23 (79%)	51 (76%)		
30%-45%	15 (19%)	10 (20%)	5 (18%)	12 (18%)		
15%-30%	4 (5%)	4 (8%)	0 (0%)	4 (6%)		
Unknown	5 (6%)	4 (8%)	1 (3%)			
MI grade						
0	28 (38%)	13 (29%)	15 (54%)	25 (38%)		
I	37 (51%)	24 (53%)	13 (46%)	33 (50%)		
II	8 (11%)	8 (18%)	0 (0%)	8 (12%)		
III	0 (0%)	0 (0%)	0 (0%)	0 (0%)		
30-d mortality		16.2%	20.8%	0 (0%)	12 (100%)	

Comparison of characteristics for patients with rescue-AV (patients needing more than 1 valve due to malposition or embolization of the first valve). P-value denotes testing within a group to study if any characteristics significantly affects 30-day mortality.

Abbreviations: AI, aortic insufficiency; BP, blood pressure; ECMO, extracorporeal membrane oxygenator; LVEF, left ventricle ejection fraction; MI, mitral insufficiency; SBP, systolic blood pressure.

∗ Comparison between Bicuspid and tricuspid.

† comparison between embolized/ectopic valve and valve in the annulus for the first valve.

Patients who had an embolized valve had a 30-day mortality of 17% (5 of 29), and patients who had the first valve anchored in the annulus had a 30-day mortality of 14% (7 of 50, *p* = 0.823, Table 4).

The characteristics that were associated with high 30-day mortality were bicuspid Sievers 0 (100% mortality), vasopressor before procedure (100% mortality), ECMO or inotropic support after the procedure (100 and 75% mortality, respectively), cardiac surgery after the procedure (100% mortality), and major stroke (71% mortality).

For the patients who survived the 30-day mark, 11% had moderate PVL, and no one had severe PVL (Table 4). Mitral insufficiency and left ventricular ejection fraction were as expected in this patient category (Table 4).

## Discussion

This retrospective study investigates the characteristics and survival of patients leaving their TAVI procedure with 2 valves due to a malpositioned or embolized first valve. A nationwide all-comers cohort spanning 6 ​years was used, and we found an incidence of 1.3% rescue-AV. The group that received a rescue-AV had a high 30-day mortality (15.2%), but after 30 days the survival curves were comparable with the control group.

One interesting finding of this study was that the rescue-AV group and control group were well-balanced in terms of demographics. The only difference found was that the rescue-AV group had a somewhat smaller AVA, while the mean aortic gradient did not differ. They also had twice the number of urgent procedures as compared to the control group. The apparent interpretation of this finding is that valve malpositioning is not directly related to patient factors, with the caveat that we did not examine the anatomy and calcium distribution in the annulus and left ventricular outflow tract. Previous studies have found that a horizontal aorta, aortic regurgitation, conical left ventricular outflow tract, bicuspid valve and, tall sinotubulur junction are predictors for valve malposition.^5^, ^6^, ^7^^,^^12^ We did not study the anatomy of the aortic root besides cuspidality and mean annular diameter, where we found no difference between the rescue-AV and control group. The occurrence of valve malpositioning is probably most dependent on factors during the procedure, such as failure to deploy at the correct annular level or pacing failure.^6^

We found a 1.3% prevalence of patients leaving the cath lab with more than 1 valve and a 15% 30-day mortality among these patients. Early reports from first-generation valves in the PARTNER I Trial showed a 2.5% frequency for rescue-AV and 1% rate of valve embolization.^13^^,^^14^ Kim et al^6^ studied the occurrence of valve embolization in a multicenter study and found a prevalence of 0.9% with an 18% 30-day mortality. Frumkin et al also studied valve embolization selectively and found an incidence of 1.4%.^5^ A recent study on an American TAVI cohort found a 2% incidence of needing a second valve with a 9.6% 30-day mortality. However, many received a second valve for a PVL indication, and only a small number in that study had an embolized valve.^7^ In the present study, 41% of the rescue-AV had an embolized first valve, which equates to a 0.5% incidence of valve embolization. All 3 previous studies found that self-expandable valves had a higher frequency of malpositioning, which is in agreement with the findings in our study. The previous studies reported a high frequency of first-generation valves while ours reported a low frequency of first-generation valves. If we analyze only the valves that are currently on the market today, we still find a 1.3% incidence of rescue-AV (Table 2). Still, more modern platforms, together with increasing operator experience, should explain the lower frequency in the present study.

One of the key findings of this study is the bimodal outcome for patients with a rescue-AV. While 30-day survival was worse than the control group, survival after the 30-day landmark was comparable to the control group, both before and after propensity score matching. Many of the early deaths were associated with other complications, such as the need for acute surgery, ECMO, or major stroke. The postprocedural echocardiogram of the survivors had a somewhat higher degree of grade I aortic insufficiency, but they were otherwise representative of a general TAVI cohort. The clinical interpretation of this finding is that if the patient had no further complications after the procedure, having had more than 1 TAVI valve implanted has little or no impact on the prognosis after 30 days until the end of follow-up (mean 880 days). To our knowledge, this report presents the longest follow-up of patients with rescue-AV.

### Limitations

This is a registry-based retrospective study that has inherent weaknesses and strengths. First, as it is registry-based, the quality of the data entry will affect results. Also, we are limited to the data that was entered, and other undocumented variables could have led to a different result. To compensate for these shortcomings, we performed a structured validation of the patients who were classified as having either 2 valves or ectopic valves. This made us reclassify 8 patients. Compared to previous reports, the use of registry data was unable to identify the cause of the first valve malpositioning. A real-world, all-comers, nationwide dataset with robust follow-up for survival is, on the other hand, a very useful tool for describing the current status of a treatment as it is not disadvantaged by the attrition seen in randomized controlled studies or limited registries.

## Conclusion

This study describes the characteristics and outcomes of patients requiring a second TAVI due to the initial TAVI valve being malpositioned or embolized. The frequency of this complication was 1.3% and was associated with a 15.2% 30-day mortality. For the patients who survived the first 30 days, subsequent outcomes were comparable to patients receiving 1 TAVI valve only.

## Ethics Statement

The study was performed in accordance with the Declaration of Helsinki.

## Funding

This work was supported by a regional government research fund under the agreement between the Swedish government and the county councils (ALF-agreement) and grants from the 10.13039/501100003793Swedish Heart-Lung Foundation. The study was approved by the National Ethical Review Board in Sweden (registration numbers 2017/995 and EPN 2019/0584).

## Impact on Daily Practice

In the rare occurrence of having to implant a second TAVI valve during the same procedure, the treating doctor wants to know how the patient will fare after the procedure. We can conclude that if there is a second serious complication, then there is a high risk for a bad short-term outcome. But if the patient survives and does well during the hospital stay, the prognosis is good.

## Disclosure Statement

HB: Consultancy and grants from Boston Scientific. AR: Consultancy and grants from Boston Scientific and Edwards Lifesciences. SJ: Consultancy from Medtronic. MS: Consultancy and advisory boards for Boston Scientific, Edwards Lifesciences, Abbott Vascular, and WL Gore. MG: Consultancy and grants from Boston Scientific. The other authors had no conflicts to declare.
