# Supplementary material for: Characteristics and Outcomes of Patients Receiving a Second Rescue Valve During Transcatheter Aortic Valve Implantation
Source: Struct Heart. 2023 Nov 14;8(2):100231. doi: 10.1016/j.shj.2023.100231 (PMC10927442; doi:10.1016/j.shj.2023.100231)

**Characteristics and outcomes for patients receiving a second bail-out valve during transcatheter aortic valve implantation.**

**Appendix 1**

**Address for Correspondence:**

Henrik Bjursten

Department of Cardiothoracic Surgery

Skåne University Hospital

221 85 Lund

Sweden

Phone: +46 46 17 53 49

Email: [henrik.bjursten@med.lu.se](mailto:henrik.bjursten@med.lu.se)

**Balancing variables for propensity score matching**

|  | Before matching | | | After matching | | |
| --- | --- | --- | --- | --- | --- | --- |
|  | Control | Rescue-2V | SMD | Control | Rescue-2V | SMD |
| Age | 81.1 ± 6.7 | 81.5 ± 7.5 | 0.047 | 82.1 ± 6.6 | 81.6 ± 7.6 | -0.112 |
| Female | 2702 (46.0%) | 36 (45.6%) | -0.047 | 64 (45.7%) | 31 (44.3%) | -0.080 |
| Serum creatinine (µmol/L) | 100.7 ± 59.6 | 96.1 ± 32.5 | -0.108 | 88.3 ± 26.7 | 95.6 ± 33.5 | -0.064 |
| Hypertension | 4526 (77.1%) | 66 (82.3%) | 0.117 | 115 (82.1%) | 57 (81.4%) | 0.012 |
| Diabetes | 1512 (25.8%) | 19 (24.1%) | -0.075 | 25 (17.9%) | 16 (22.9%) | -0.008 |
| Peripheral vascular disease | 724 (12.3%) | 12 (15.2%) | 0.103 | 20 (14.3%) | 11 (15.7%) | -0.053 |
| NYHA class IV | 571 (9.7%) | 10 (12.6%) | 0.075 | 19 (13.6%) | 9 (12.9%) | 0.001 |
| LVEFseverly sepressed | 421 (7.2%) | 8 (10.1%) | 0.095 | 15 (10.7%) | 7 (10%) | -0.162 |
| Urgent procedure | 663 (11.3%) | 16 (20.3%) | 0.279 | 24 (17.1%) | 15 (21.4%) | 0.007 |
| Bicuspid valve | 444 (7.6%) | 9 (12.1%) | 0.091 | 16 (11.4%) | 7 (10%) | 0.128 |
| Aortic valve area (mm2) | 0.71 ± 0.16 | 0.64 ± 0.15 | -0.362 | 0.62 ± 0.2 | 0.63 ± 0.2 | 0.024 |

SMD = standardized mean difference

**Balancing plot before and after matching**


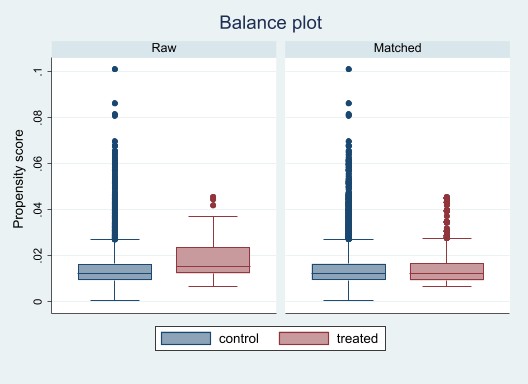

Supplement: Supplemental Material [file mmc1.docx]
